# Creep in Primary Consolidation with Rate of Loading Approach

**DOI:** 10.1038/s41598-019-45498-0

**Published:** 2019-06-20

**Authors:** Gang Bi, Shua Ni, Dong Wang, Yeqiang Chen, Jianfei Wei, Wenzong Gong

**Affiliations:** 1 Department of Civil Engineering, Yango University, Fuzhou, Fujian, 350015 China; 2 Department of Civil Engineering, Johns Hopkins University, Baltimore, MD 21218 USA; 3 Department of Civil Engineering, University of Wollongong, New South Wales, 2522 Australia; 4 Guangxi Transportation Research & Consulting Co, Nanning, Guangxi 530007 China; 5 Guangxi Communications Design Group Co, Nanning, Guangxi 530012 China

**Keywords:** Civil engineering, Applied mathematics

## Abstract

The debate on creep in primary consolidation is analysed with a power law model following an approach in which creep is considered as rate of loading. According to this approach, primary consolidation is one type of rate of loading. To verify this approach, two types of tests, standard oedometer test and oedometer test with drainage prevented, are conducted on three types of soils (two from NGES and the other from Port of Guangzhou). The result: creep exponents obtained from two kinds of tests agree well with each other. Moreover, the approach is further validated by tracking, for over 80 years, the data from settlement of the case history San Jacinto Monument, which is inconsistent with data calculated from the classical method. In the end, procedure of this approach, with which long term settlement is predicted, is illustrated, and this approach is compared with the classical method.

## Introduction

The long term settlement of soil includes two types of time dependent behavior: consolidation and creep. According to Terzaghi^1^, consolidation is “any process which involves a decrease in water content of saturated soil without replacement of water by air”, and in the classical method developed by Terzaghi, soils are tested with an oedometer test which defined a separation between primary consolidation and secondary compression, the latter of which is thought to be caused by creep.

However, a debate has lasted several decades that whether there is creep in primary consolidation. On one hand, Terzaghi defined creep as the continuous deformation under constant effective stress, and plenty of studies on creep behavior of various soils by oedometer test have been based on this definition^2–4^; on the other hand, other researchers^5–11^ proposed creep could be taken into consideration in the whole consolidation process, as the mechanism^12^ governing the creep behavior should not change between primary consolidation and secondary compression. The latter statement is further supported by creep research on sands by oedometer test^13–16^.

The assumption of constant stress is the crucial condition. The study of creep behavior is often conducted under constant (total) stress in various fields of science and engineering. The initial definition of creep, however, in materials science, is particles and the structure of a solid material slowly adjusting to resist the applied external stress in the long term. This definition has never explicitly and implicitly stated that creep should be under constant stress. In this article, creep is considered as a time dependent deformation, regardless whether the applied stress is constant or not.

Lab test performed by Casagrande and Wilson^17^ in Massachusetts Institute of Technology (MIT) on clays and shales, first studied creep as rate of loading. They found that the ultimate strength is significantly lower than results of tests with slower rate of loading: the slower the rate of loading, the lower of the ultimate strength obtained.

**Figure 1:**
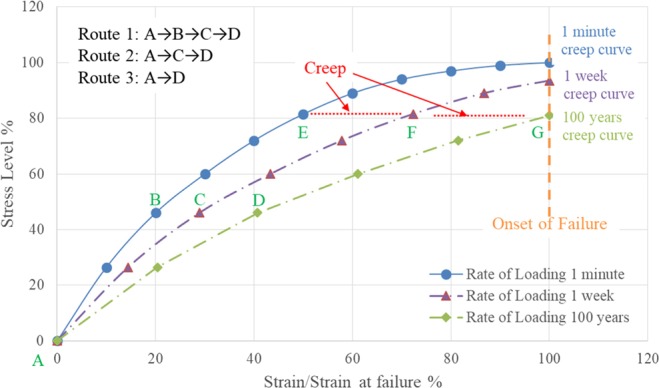
Creep and rate of loading.

This article uses the approach “creep as rate of loading” on a clay^18,19^ with an illustrated example (Fig. 1) to inquire into the relationship between creep and rate of loading.

In Fig. 1, the curve with the legend of “rate of loading 1 minute” represents tests without creep. Standard UU triaxial shear test, e.g., will take about 20 minutes, which is so short that creep deformation is negligible; the curve with the legend of “rate of loading 1 week” represents the creep study performed by Casagrande and Wilson^17^; the curve with the legend of “rate of loading 100 years” represent an extreme case as the design life of most structures is 50 years or 100 years.

Three routes are introduced in Fig. 1, namely, Route 1 (A → B → C → D), Route 2 (A → C → D) and Route 3 (A → D). Under Route 1 with a fast rate of loading (1 minute), a soil loads from point A to reach point B, then creeps 1 week to reach point C, and then continues to creep (slightly less than) 100 years to reach point D. Under Route 2 with a quite slow rate of loading (1 week), a soil loads from point A to reach point C, and then continues to creep (slightly less than) 100 years to reach point D. Under Route 3 with a tremendously slow rate of loading (100 years), a soil loads from point A to reach point D directly.

The comparison showed the existence of creep in Route 2 and Route 3. It is worth mentioning that the primary consolidation of 1D consolidation test is similar to Route 2, also one type of rate of loading (i.e., effective stress is slowly increasing).

On the other way, curves of “rate of loading” are also called creep curves. For instance, the curve of “rate of loading 100 years” will be “100 years creep curve”, which has been demonstrated by the author in other publications on creep^18,19^.

The objective of this study is to scrutinize the debate “creep in primary consolidation” by a power law model with the approach “creep as rate of loading”. Specifically, this study will (i) distinguish creep from primary consolidation with two kinds of oedometer tests, (ii) further demonstrate the approach with a case history with over 80 year’s settlement data, and (iii) predict long term settlement in engineering practice with comparsion to the classical method.

## Methods

A power law model was proposed by Briaud and Garland^20^ to describe the rate of loading behavior on piles in clay. It has been extended by the author to present the time dependent behavior of various sources of soils on diversified data^18^. The model is written as below:

1 $$\frac{s}{{s}_{1}}={(\frac{t}{{t}_{1}})}^{n}$$

where, the deformation (settlement, strain, etc.) s_1_ is usually chosen to be the value of deformation *s* observed at reference time t_1_, which could be any chosen time, such as 1 second, 1 minute, 1 day … *et al*., and *n* is the viscous exponent.

It has been demonstrated by various sources of data on diverse soils^18,19^ that the proposed model will produce a remarkably straight line, if only creep deformation is included, and the slope of the line will be viscous exponent.

Under standard oedometer test, the proposed model produces a bilinear strain-time curve in the log-log plot. A special oedometer test with drainage prevented is conducted, under which the proposed model only produces a straight line in the log-log plot, since it only includes creep deformation, excluding deformation due to excess pore puressure dissipation (namely, one straight line with the proposed model applied). It is assumed that creep exponents from both types of tests will be very close, if not identical. The validation of this assumption can demonstrate that the approach “creep as rate of loading” works well for primary consolidation (i.e., there is creep in primary consolidation).

**Figure 2:**
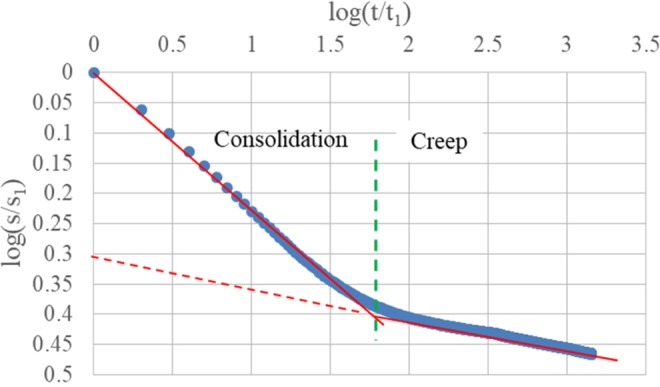
Oedometer test.

By this assumption, a bilinear curve is presented and the line with the lower slope could be extended to primary consolidation (Fig. 2).

The slope of the first line (primary consolidation) equals to *n* value including both consolidation and creep (n_both_), while the slope of the second line (secondary compression) equals to *n* value only including creep (n_cr_). If the angle corresponding to n_both_ is *α*, and the angle corresponding to n_cr_ is *β*, the angle of slope for the *n* value only including consolidation (n_con_) during the primary consolidation will be (*α* − *β*). The *n* value n_con_ can be calculated by Equation 2.

2 $${n}_{con}=\,\tan (\alpha -\beta )=\frac{\tan (\alpha )-\,\tan (\beta )}{1+\,\tan (\alpha )\ast \tan (\beta )}=\frac{{n}_{both}-{n}_{cr}}{1+{n}_{both}\ast {n}_{cr}}$$

while in general n_both_ is quite small and n_cr_ is significantly less than n_both_, Equation 2 can be simplified as:

3 $${n}_{con}=\frac{{n}_{both}-{n}_{cr}}{1+{n}_{both}\ast {n}_{cr}}\approx {n}_{both}-{n}_{cr}$$

## Results

### Oedometer tests

Oedometer tests are performed on soils (two clays and one sand) from different locations to verify the assumption made above. Clay 1 is soft clay from Port of Guangzhou, and its long term settlement is a big concern for the project, for which an accurate prediction of the settlement will be very helpful. Soil properties of this clay are summarized in Table 1.

**Table 1:** Soil Property of Clay 1 from Port of Guangzhou.

Sample Depth (m)	Water content (%)	Density (g/cm^3^)	Saturation (%)	Liquid Limit (%)	Plastic Limit (%)	Cohesion (kPa)	Friction Angle (deg)
9.0	47.7	1.75	100	44	27	23.1	4.5

Note: strength parameters are per quick direct shear test.

**Figure 3:**
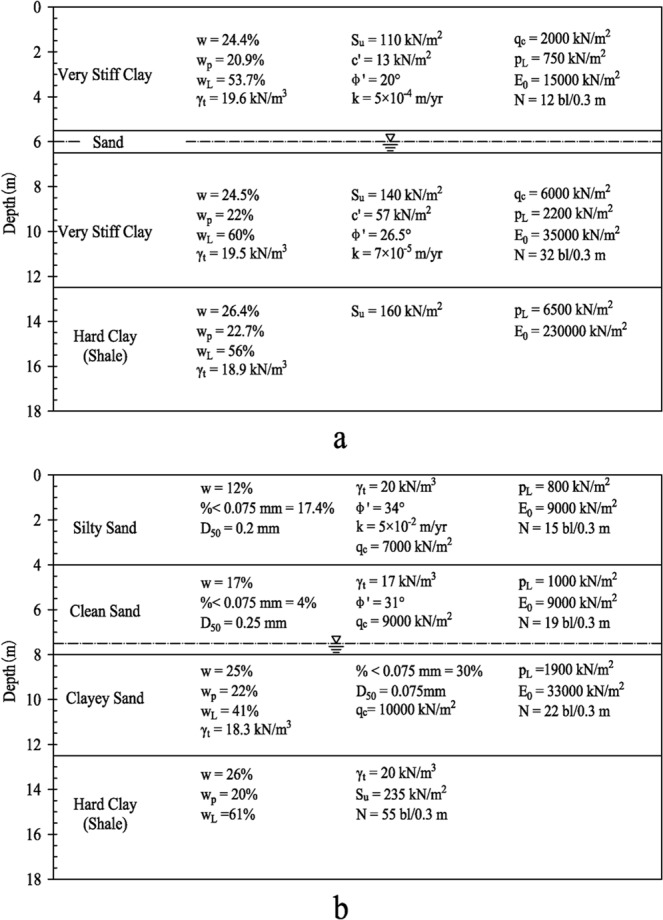
Soil properties at NGES: (**a**) clay; (**b**) sand^21^.

Clay 2 and sand are soils located National Geotechnical Experimentation Site (NGES) at Texas A&M University. Soil samples are obtained at depth from 2 m to 6 m beneath the ground. A summary of soil properties (Fig. 3) at NGES is reported by Briaud^21^. Oedometer test is performed on sand to demonstrate that the approach is suitable for various types of soils.

**Figure 4:**
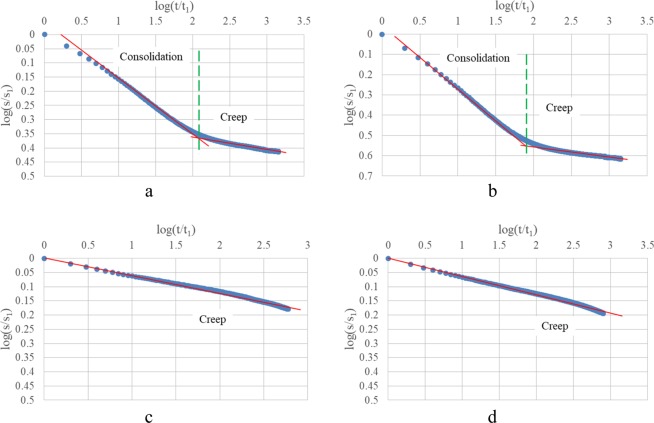
Clay 1: (**a**,**b**) Oedometer test 125 kPa, 1000 kPa; (**c**,**d**) Oedometer test with drainage prevented, 125 kPa, 1000 kPa.

Strain-time curves of oedometer test and oedometer test with drainage prevented performed on clay 1 under the applied vertical stress 125 kPa and 1000 kPa are presented in Fig. 4, in which strain-time curves show a clear bilinear trend in log-log plots with lines corresponding to primary consolidation and secondary compression respectively. In contrast, only a relative linear trend in log-log plots is there for oedometer test with drainage prevented corresponding to creep.

**Figure 5:**
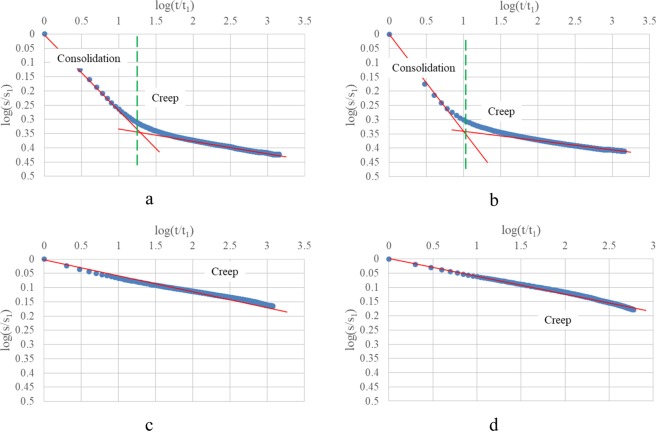
Clay 2: (**a,b**) Oedometer test 125 kPa, 1000 kPa; (**c,d**) Oedometer test with drainage prevented, 125 kPa, 1000 kPa.

Strain-time curves of oedometer test and oedometer test with drainage prevented performed on clay 2 under the applied vertical stress 125 kPa and 1000 kPa are presented in Fig. 5. Observations from Fig. 5 are the same to those from Fig. 4.

**Figure 6:**
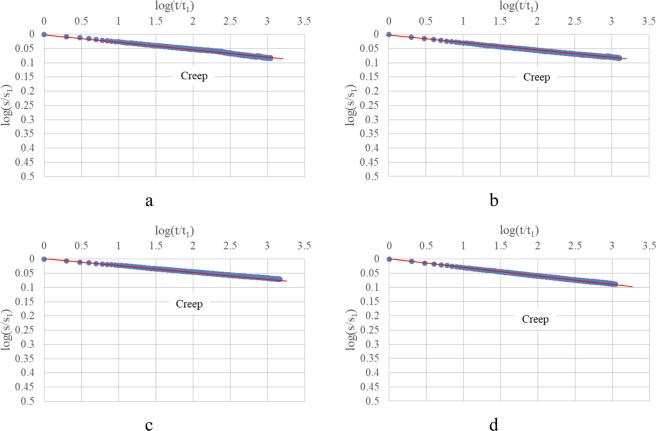
Sand: (**a**,**b**) Oedometer test 125 kPa, 1000 kPa; (**c**,**d**) Oedometer test with drainage prevented, 125 kPa, 1000 kPa.

**Figure 7:**
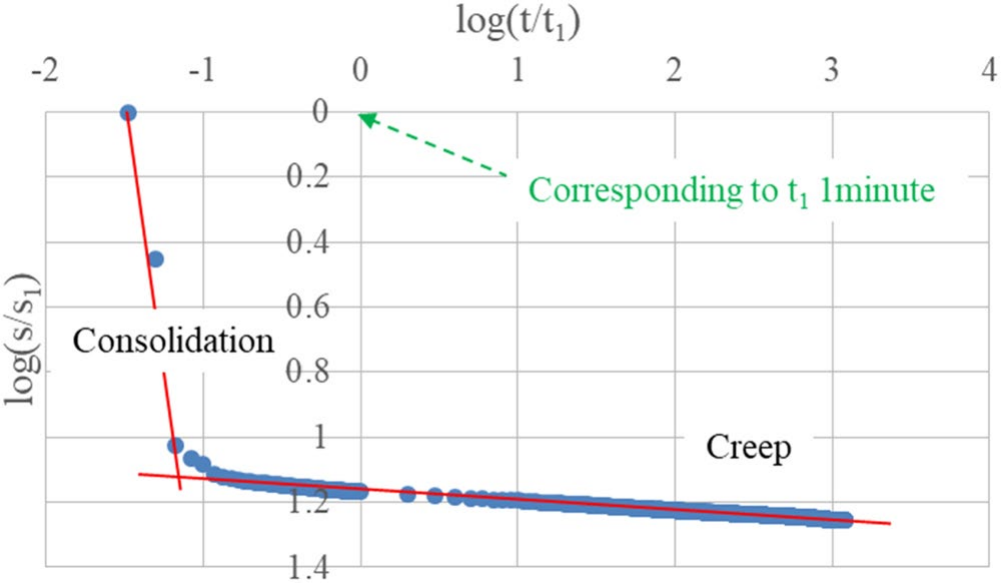
Oedometer test on sand with record data per second.

Strain-time curves of oedometer test and oedometer test with drainage prevented performed on sand under the applied vertical stress 125 kPa and 1000 kPa are presented in Fig. 6. Due to the fact that the primary consolidation for sand will take only a few seconds, and the reference time t_1_ is 1 minute as the minimum frequency to record data is per minute, strain-time curves show a linear trend in log-log plots from regardless oedometer test or oedometer test with drainage prevented. To deal with this problem, one additional oedometer test on sand is conducted with a higher frequency to record data per second (compared to the standard frequency one data per minute). In this case, the primary consolidation becomes significant (Fig. 7) and strain-time curves are characterized by a linear trend which is similar to the trend for clays.

**Figure 8:**
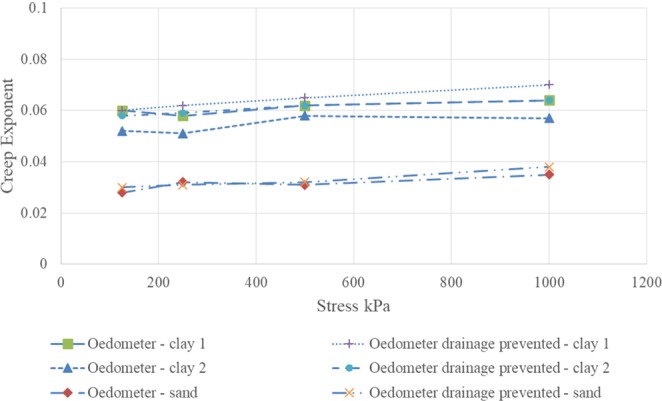
Creep exponent from two types of oedometer tests.

Slopes (n_cr_) from oedometer test and oedometer test with drainage prevented for soils tested above are compared in Fig. 8, which showed that n_cr_ from both kinds of tests is very close, if not equal, validating the previous assumption of the approach “creep as rate of loading”.

It is worth mentioning that n_cr_ from both kinds of tests are essentially independent of stress. This result agrees with the finding on several data from other sources (such as, load tests on spread footings, soil nail pullout tests, and UU creep triaxial tests) investigated by the author^18,19^.

### Case history

A special case history with over 80 years’ record data of settlement is introduced herein to further demonstrate the approach.

**Figure 9:**
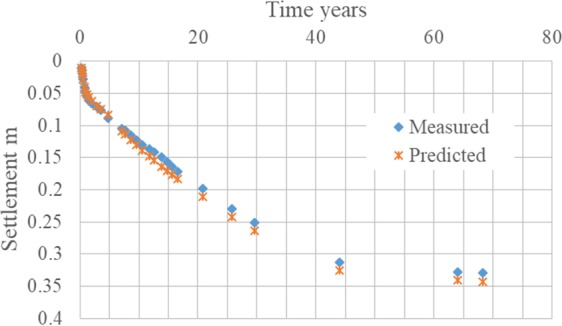
Actual versus predicted settlement, San Jacinto monument.

Briaud *et al*.^22,23^ present a valuable case history, i.e., the San Jacinto Monument, which was built in 1936 for celebrating Texas joining in United States 100 years and its settlement has been recorded since then. The prediction of the settlement illustrated by Briaud *et al*.^22,23^ by several methods including classical method, PMT, CPT, is unsatisfactory. By comparison, the prediction with the approach in this article agrees well with the actual data (Fig. 9).

**Figure 10:**
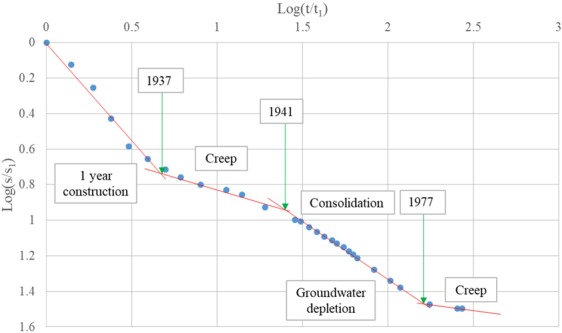
Settlement-time curve of San Jacinto monument, with the approach.

The settlement by the proposed approach will transform to four line segments in the log-log plot (Fig. 10) with each line segment illustrated with the approach. For example, the first line segment corresponding to the 1 year construction, is a type of rate of loading as the net pressure is increasing. The third line segment matching well with the groundwater depletion in Houston area^24^, is consolidation which has been demonstrated as one type of rate of loading.

In conclusion, compared to the classical method, the approach with the power law model is more flexible and accurate to predict the long term (several decades) settlement for soils in engineering practice.

### Prediction

Besides being capable of describing the long term time dependent behavior for soils, this approach has another advantage of including only a few parameters that are easy to obtain.

With this approach, an example is presented to illustrate the prediction which is also compared to the prediction by the classical method. The thickness of a soil layer in the field is 1.9 m (100 times the thickness in 1D consolidation test), and the soil is assumed to be identical to clay 2 in this article. The above and below layer of the analyzed soil layer are both permeable. The soil is subject to a stress increment from 250 kPa to 500 kPa.

According to the classical method, the final increment of settlement (also strain) because of the stress increment from 250 kPa to 500 kPa is calculated with equations below:

4 $$\begin{array}{c}{\rm{\Delta }}\varepsilon =\frac{{e}_{1}-{e}_{2}}{1+{e}_{0}}\\ {\rm{\Delta }}{H}_{field}=\frac{{e}_{1}-{e}_{2}}{1+{e}_{0}}{H}_{field}\end{array}$$

where,

*e*_1_ *e*_2_: Void ratio corresponding to 250 kPa and 500 kPa, respectively, obtained from e-logP curve based on oedometer test in the lab;

*e*_0_: Initial void ratio, obtained from e-logP curve based on oedometer test in the lab;

*H*_*field*_: Thickness of soil layer in the field, 1.9 m herein;

Δ*H*_*field*_: Final increment of settlement;

Δ*ε*: Final increment of strain;

The average degree of consolidation in the soil layer is approximated with Equation 5, when the value is no less than 30%:

5 $$\bar{U}=1-\frac{8}{{\pi }^{2}}\exp (-\frac{{\pi }^{2}}{4}\frac{{c}_{v}\cdot t}{{H}_{field}^{2}})$$

where,

$\bar{U}$: Average degree of consolidation;

*H*_*field*_: Length of drainage path in the field, which is half of thickness of soil layer in the field, 0.95 m in this example;

*c*_*v*_: Coefficient of consolidation, obtained from strain-time (semi-log scales) curve based on oedometer test in the lab;

Combining Eqs 4 and 5, will yield the long-term deformation prediction by the classical method (Fig. 11).

**Figure 11:**
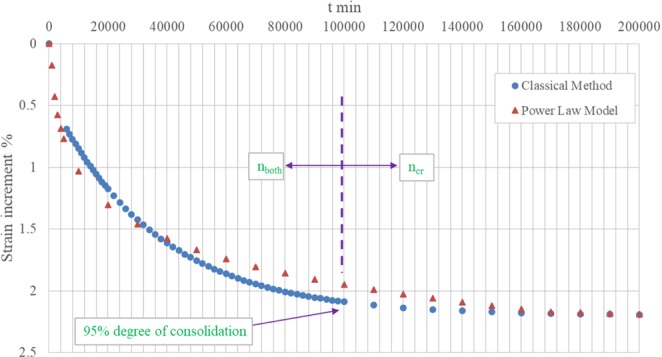
Prediction by classical method vs. model.

The prediction with the power law model with the rate of loading approach will follow:

6 $$\begin{array}{ll}\frac{s}{{s}_{1}}={(\frac{t}{{t}_{1}})}^{{n}_{both}} & t\le {t}_{eop}\\ \frac{s}{{s}_{1}}={(\frac{t}{{t}_{eop}})}^{{n}_{cr}}{(\frac{{t}_{eop}}{{t}_{1}})}^{{n}_{both}} & t\ge {t}_{eop}\end{array}$$

where,

*t*_*eop*_: Time corresponding to end of primary consolidation. It is taken as the time corresponding to 95% degree of consolidation according to the classical method.

In order to predict the settlement on field site according to oedometer test in the lab,

While,

7 $$\frac{{({t}_{eop})}_{field}}{{({t}_{eop})}_{lab}}=\frac{\frac{{({T}_{v})}_{field}\cdot {H}_{field}^{2}}{{({c}_{v})}_{field}}}{\frac{{({T}_{v})}_{lab}\cdot {H}_{lab}^{2}}{{({c}_{v})}_{lab}}}={(\frac{{H}_{field}}{{H}_{lab}})}^{2}\cdot \frac{{({c}_{v})}_{lab}}{{({c}_{v})}_{field}}\cdot \frac{{({T}_{v})}_{field}}{{({T}_{v})}_{lab}}=K\cdot {(\frac{{H}_{field}}{{H}_{lab}})}^{2}$$

In this example,

8 $$\frac{{({t}_{eop})}_{field}}{{({t}_{eop})}_{lab}}=K\cdot {(\frac{{H}_{field}}{{H}_{lab}})}^{2}=1\cdot {(\frac{1.9m/2}{1.9cm/2})}^{2}=10000$$

where,

(*t*_*eop*_)_*lab*_: Time corresponding to end of primary consolidation in the lab;

(*t*_*eop*_)_*field*_: Time corresponding to end of primary consolidation in the field site;

*H*_*field*_: Length of drainage path in the field, in this example 0.95 m, which is half of thickness of soil layer in the field;

*H*_*lab*_: Length of drainage path in the lab, in this paper 0.95 cm, which is half of thickness of specimen in 1D consolidation test;

t_eop_ is referring to t_1_:

9 $${({t}_{1})}_{field}={({t}_{1})}_{lab}\cdot \frac{{({t}_{eop})}_{field}}{{({t}_{eop})}_{lab}}=10000\,\min \,\approx 1w$$

Assuming:

10 $$\begin{array}{c}{({n}_{both})}_{field}={({n}_{both})}_{lab}\\ {({n}_{cr})}_{field}={({n}_{cr})}_{lab}\end{array}$$

The long-term deformation prediction in the field site will yield (Fig. 11). The prediction based on classical method and that based on the power law model with the rate of loading approach agree well with each other, which demonstrates that the proposed power law model with the rate of loading approach is feasible in the engineering practice.

## Discussion

The approach in which creep is considered as rate of loading by Casagrande and Wilson^17^ is reintroduced to study whether there is creep in primary consolidation. A power law model is proposed to describe the rate of loading behavior of soils in this paper.

Since creep is defined as the solid material slowly deforming to resist the applied external stress in the long term, creep is one type of time dependent behavior, which is ongoing all the time, regardless the change of the applied external stress. However, the rate of change of the applied external stress. However, the rate of change of the applied external stress (rate of loading) will have an impact on the magnitude of slow deformation due to creep. Thus, the proposed power law model is able to describe the approach “creep as rate of loading”, and the exponent in the model is the viscous exponent which represents the mechanism of the time dependent behavior (including creep) of a solid material under the applied external stress.

An assumption is made that primary consolidation is one type of rate of loading; the proposed power law model can well predict deformations of primary consolidation, including both consolidation and creep. Moreover, the model can isolate and represent the creep from deformations of primary consolidation. The mechanism of creep behavior in primary consolidation of oedometer test will be identical to that in secondary compression of oedometer test, to that in primary consolidation of oedometer test with drainage prevented and to that in secondary compression of oedometer test with drainage prevented. The mechanism could also be represented by the power law model. In this case, creep is isolated from consolidation in primary consolidation of oedometer test.

Standard oedometer tests and oedometer tests with drainage prevented, performed on three soils (two from NGES and the other from Port of Guangzhou) have validated the assumption, and results of creep exponent obtained from two types of tests are very close. The approach is further strengthened by the data from settlement of the case history San Jacinto Monument, for over 80 years, while the result poorly match the prediction by the classical method. The rate of loading approach with the power law model is more flexible and accurate than the classical method in predicting the long term (several decades) settlement for soils in engineering practice. The procedure to make the prediction in practice with the approach and the proposed model is illustrated in detail with a simple example both in the laboratory and in the field site. The procedure is also compared with the classical method.

## Data Availability

The datasets generated during and/or analysed during the current study are available from the corresponding author on reasonable request.
