# Author Correction: Creep in Primary Consolidation with Rate of Loading Approach

**DOI:** 10.1038/s41598-020-59786-7

**Published:** 2020-02-13

**Authors:** Gang Bi, Shuna Ni, Dong Wang, Yeqiang Chen, Jianfei Wei, Wenzong Gong

**Affiliations:** 1 Department of Civil Engineering, Yango University, Fuzhou, Fujian 350015 China; 2 Department of Civil Engineering, Johns Hopkins University, Baltimore, MD 21218 USA; 3 Department of Civil Engineering, University of Wollongong, New South Wales, 2522 Australia; 4 Guangxi Transportation Research & Consulting Co, Nanning, Guangxi 530007 China; 5 Guangxi Communications Design Group Co, Nanning, Guangxi 530012 China

Correction to: *Scientific Reports* 10.1038/s41598-019-45498-0, published online 20 June 2019

The original version of this Article contained a typographical error in the spelling of the author Shuna Ni, which was incorrectly given as Shua Ni. This has now been corrected in the PDF and HTML versions of the Article.

